# *N*-(3-Methyl­phen­yl)quinoxalin-2-amine monohydrate

**DOI:** 10.1107/S1600536810031260

**Published:** 2010-08-11

**Authors:** Azila Idris, Zanariah Abdullah, Azahar Ariffin, Zainal A. Fairuz, Seik Weng Ng, Edward R. T. Tiekink

**Affiliations:** a Department of Chemistry, University of Malaya, 50603 Kuala Lumpur, Malaysia

## Abstract

The quinoxaline system in the title hydrate, C_15_H_13_N_3_·H_2_O, is roughly planar, the r.m.s. deviation for the 18 non-H atoms being 0.188 Å; this conformation features a short intra­molecular C—H⋯N(pyrazine) inter­action. In the crystal, the amine H atom forms an N—H⋯O hydrogen bond to the water mol­ecule, which in turn forms two O—H⋯N hydrogen bonds to the pyrazine N atoms of different organic mol­ecules. These inter­actions lead to supra­molecular arrays in the *bc* plane that are two mol­ecules thick; additional π–π inter­actions stabilize the layers [ring centroid–centroid distance = 3.5923 (7) Å]. The layers stack along the *a*-axis direction *via* C—H⋯π contacts.

## Related literature

For a related structure, see: Fairuz *et al.* (2010 ▶). For background to the fluorescence properties of compounds related to the title compound, see: Kawai *et al.* (2001 ▶); Abdullah (2005 ▶).
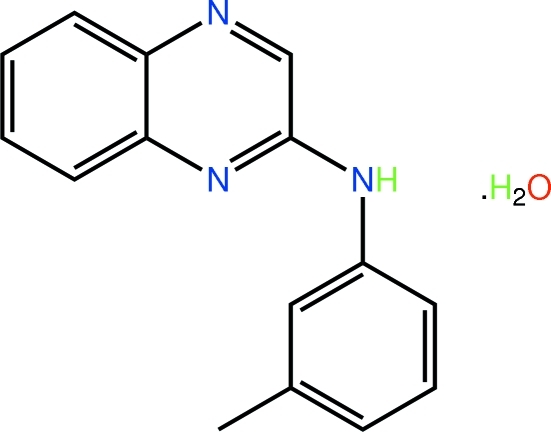


## Experimental

### 

#### Crystal data

C_15_H_13_N_3_·H_2_O
*M* *_r_* = 253.30
Monoclinic, 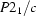

*a* = 10.9002 (8) Å
*b* = 11.1048 (8) Å
*c* = 11.1715 (8) Å
β = 106.780 (1)°
*V* = 1294.67 (16) Å^3^
*Z* = 4
Mo *K*α radiation
μ = 0.08 mm^−1^
*T* = 100 K
0.30 × 0.20 × 0.05 mm

#### Data collection

Bruker SMART APEX CCD diffractometer
Absorption correction: multi-scan (*SADABS*; Sheldrick, 1996 ▶) *T*_min_ = 0.942, *T*_max_ = 1.000
12521 measured reflections
3100 independent reflections
2608 reflections with *I* > 2σ(*I*)
*R*_int_ = 0.028

#### Refinement

*R*[*F* ^2^ > 2σ(*F* ^2^)] = 0.039
*wR*(*F* ^2^) = 0.110
*S* = 1.02
3100 reflections
185 parameters
3 restraints
H atoms treated by a mixture of independent and constrained refinement
Δρ_max_ = 0.24 e Å^−3^
Δρ_min_ = −0.27 e Å^−3^

### 

Data collection: *APEX2* (Bruker, 2009 ▶); cell refinement: *SAINT* (Bruker, 2009 ▶); data reduction: *SAINT*; program(s) used to solve structure: *SHELXS97* (Sheldrick, 2008 ▶); program(s) used to refine structure: *SHELXL97* (Sheldrick, 2008 ▶); molecular graphics: *ORTEP-3* (Farrugia, 1997 ▶) and *DIAMOND* (Brandenburg, 2006 ▶); software used to prepare material for publication: *publCIF* (Westrip, 2010 ▶).

## Supplementary Material

Crystal structure: contains datablocks global, I. DOI: 10.1107/S1600536810031260/hb5601sup1.cif

Structure factors: contains datablocks I. DOI: 10.1107/S1600536810031260/hb5601Isup2.hkl

Additional supplementary materials:  crystallographic information; 3D view; checkCIF report

## Figures and Tables

**Table 1:** Hydrogen-bond geometry (Å, °) *Cg*1 is the centroid of the C10–C15 ring.

*D*—H⋯*A*	*D*—H	H⋯*A*	*D*⋯*A*	*D*—H⋯*A*
C6—H6⋯N2	0.95	2.34	2.9482 (14)	122
N1—H1*n*⋯O1w	0.87 (1)	2.03 (1)	2.8951 (12)	176 (2)
O1w—H1*w*⋯N2^i^	0.85 (1)	2.13 (1)	2.9382 (13)	160 (2)
O1w—H2*w*⋯N3^ii^	0.84 (1)	2.15 (1)	2.9504 (12)	158 (2)
C7—H7b⋯*Cg*1^iii^	0.98	2.71	3.6532 (14)	161

Symmetry codes: (i) 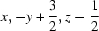; (ii) 
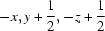
; (iii) 
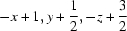
.

**Fig. 1.**
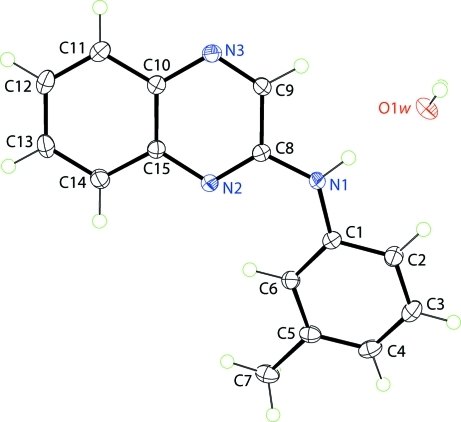
The molecular structure of (I) showing displacement ellipsoids at the 50% probability level.

**Fig. 2.**
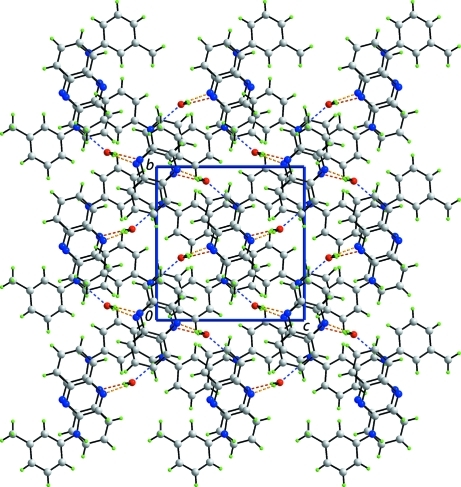
Supramolecular layer in (I) in the bc plane mediated by O–H···N and N–H···O hydrogen bonds, shown as orange and blue dashed lines, respectively.

**Fig. 3.**
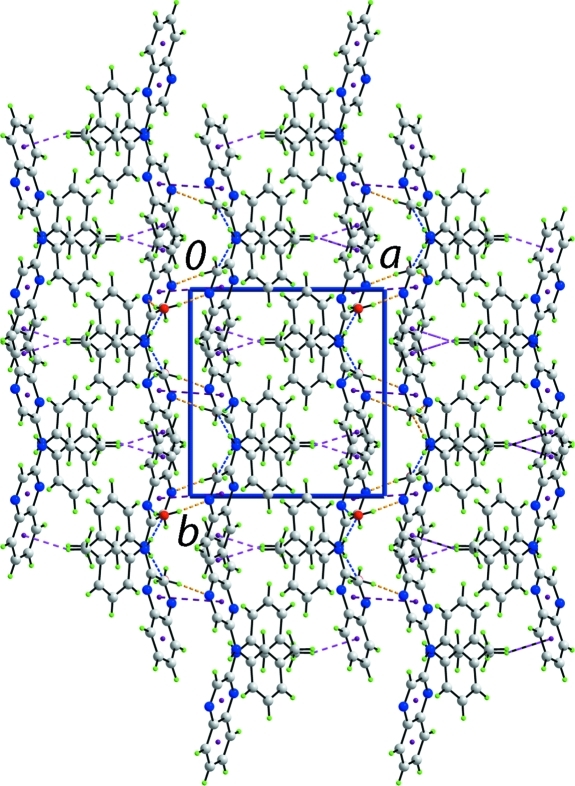
Unit-cell contents shown in projection down the c axis in (I), highlighting the stacking of layers. The O–H···N, N–H···O, C–H···π and π–π interactions are shown as orange, blue, pink and purple dashed lines, respectively.

## supplementary crystallographic information

### Comment

The title hydrate, (I), was investigated in continuation of studies (Fairuz *et al.*, 2010) into molecules that present interesting fluorescence properties (Kawai *et al.* 2001; Abdullah, 2005). The asymmetric unit of (I), Fig. 1, comprises a molecule of *N*-(3-methylphenyl)quinoxalin-2-amine and a water molecule of crystallization. The organic molecule is essentially planar with the r.m.s. deviation of the 18 non-hydrogen atoms being 0.188 Å [maximum deviations = 0.358 (1) Å for atom C7 and -0.243 (1) Å for C2]. The greatest twists in the molecule occur about the *N*(amine)–C bonds with the values of the C1–N1–C8–N2 and C8–N1–C1–C6 torsion angles being 9.51 (18) and 8.48 (18) °, respectively. An intramolecular C–H···N2 contact, Table 1, contributes to the stability of the almost planar arrangement. The latter association does not preclude this pyrazine-N atom from participating in an intermolecular interaction. The amine forms a N–H···O hydrogen bond to the water molecule and each water-H forms a O–H···N hydrogen to a pyrazine-N of different molecules, Table 1. The result of this is the formation of layers two molecules thick, Fig. 2. Layers are further stabilized by π–π interactions occurring between centrosymmetrically related pyrazine rings [ring..centroid···centroid distance = 3.5923 (7) Å for symmetry operation -*x*, 1 - *y*, 1 - *z*]. Layers are inter-digitated along the *a* axis, Fig. 3, with the primary connections between them being of the type C–H···O, Table 1.

### Experimental

2-Chloroquinoxaline (0.3260 g, 0.002 mol) dissolved in ethanol (5 ml) was added to *m*-toluidine (0.21 ml, 0.002 mol). The mixture refluxed for 5 h and extracted with chloroform (3 × 10 ml). Evaporation of solvent gave the crude product and pure 2-*N*-(*m*-methyl)anilinoquinoxaline was obtained after separating using column chromatography with EtOAc:hexane (1:3) as the eluent. Recrystallization from its ethanol solution yield colorless prisms of (I) after few days.

### Refinement

Carbon-bound H-atoms were placed in calculated positions (C—H 0.95 to 0.98 Å) and were included in the refinement in the riding model approximation, with *U*_iso_(H) set to 1.2 to 1.5*U*_equiv_(C). The O– and N-bound H-atoms were located in a difference Fourier map, and were refined with distance restraints of O–H = 0.84±0.01 Å and N–H 0.86±0.01 Å, respectively; the *U*_iso_ values were freely refined.

### Crystal data

**Table:**

C_15_H_13_N_3_·H_2_O	*F*(000) = 536
*M**_r_* = 253.30	*D*_x_ = 1.299 Mg m^−^^3^
Monoclinic, *P*2_1_/*c*	Mo *K*α radiation, λ = 0.71073 Å
Hall symbol: -P 2ybc	Cell parameters from 4286 reflections
*a* = 10.9002 (8) Å	θ = 2.7–28.1°
*b* = 11.1048 (8) Å	µ = 0.08 mm^−^^1^
*c* = 11.1715 (8) Å	*T* = 100 K
β = 106.780 (1)°	Prism, colourless
*V* = 1294.67 (16) Å^3^	0.30 × 0.20 × 0.05 mm
*Z* = 4	

### Data collection

**Table:**

Bruker SMART APEX CCD diffractometer	3100 independent reflections
Radiation source: fine-focus sealed tube	2608 reflections with *I* > 2σ(*I*)
graphite	*R*_int_ = 0.028
ω scans	θ_max_ = 27.5°, θ_min_ = 1.9°
Absorption correction: multi-scan (*SADABS*; Sheldrick, 1996)	*h* = −14→14
*T*_min_ = 0.942, *T*_max_ = 1.000	*k* = −14→14
12521 measured reflections	*l* = −13→14

### Refinement

**Table:**

Refinement on *F*^2^	Primary atom site location: structure-invariant direct methods
Least-squares matrix: full	Secondary atom site location: difference Fourier map
*R*[*F*^2^ > 2σ(*F*^2^)] = 0.039	Hydrogen site location: inferred from neighbouring sites
*wR*(*F*^2^) = 0.110	H atoms treated by a mixture of independent and constrained refinement
*S* = 1.02	*w* = 1/[σ^2^(*F*_o_^2^) + (0.0605*P*)^2^ + 0.3508*P*] where *P* = (*F*_o_^2^ + 2*F*_c_^2^)/3
3100 reflections	(Δ/σ)_max_ < 0.001
185 parameters	Δρ_max_ = 0.24 e Å^−^^3^
3 restraints	Δρ_min_ = −0.27 e Å^−^^3^

### Special details

**Table:**

Geometry. All e.s.d.'s (except the e.s.d. in the dihedral angle between two l.s. planes) are estimated using the full covariance matrix. The cell e.s.d.'s are taken into account individually in the estimation of e.s.d.'s in distances, angles and torsion angles; correlations between e.s.d.'s in cell parameters are only used when they are defined by crystal symmetry. An approximate (isotropic) treatment of cell e.s.d.'s is used for estimating e.s.d.'s involving l.s. planes.
Refinement. Refinement of *F*^2^ against ALL reflections. The weighted *R*-factor *wR* and goodness of fit *S* are based on *F*^2^, conventional *R*-factors *R* are based on *F*, with *F* set to zero for negative *F*^2^. The threshold expression of *F*^2^ > σ(*F*^2^) is used only for calculating *R*-factors(gt) *etc*. and is not relevant to the choice of reflections for refinement. *R*-factors based on *F*^2^ are statistically about twice as large as those based on *F*, and *R*- factors based on ALL data will be even larger.

### Fractional atomic coordinates and isotropic or equivalent isotropic displacement parameters (Å^2^)

**Table:**

	*x*	*y*	*z*	*U*_iso_*/*U*_eq_	
O1W	0.13500 (8)	0.90644 (8)	0.33421 (8)	0.0223 (2)	
N1	0.23584 (9)	0.74212 (8)	0.54040 (9)	0.0164 (2)	
N2	0.22297 (8)	0.54565 (8)	0.61373 (8)	0.0155 (2)	
N3	0.09733 (9)	0.48107 (8)	0.36216 (9)	0.0173 (2)	
C1	0.31138 (10)	0.80081 (10)	0.64828 (10)	0.0160 (2)	
C2	0.32032 (11)	0.92624 (10)	0.63975 (11)	0.0197 (2)	
H2	0.2719	0.9673	0.5668	0.024*	
C3	0.40005 (11)	0.99008 (11)	0.73821 (12)	0.0229 (3)	
H3	0.4062	1.0751	0.7323	0.027*	
C4	0.47106 (11)	0.93112 (11)	0.84546 (11)	0.0221 (3)	
H4	0.5267	0.9758	0.9117	0.027*	
C5	0.46125 (10)	0.80690 (11)	0.85650 (10)	0.0190 (2)	
C6	0.38116 (10)	0.74173 (10)	0.75738 (10)	0.0171 (2)	
H6	0.3741	0.6569	0.7641	0.021*	
C7	0.53811 (11)	0.74244 (12)	0.97319 (11)	0.0241 (3)	
H7A	0.5289	0.7848	1.0471	0.036*	
H7B	0.6286	0.7410	0.9755	0.036*	
H7C	0.5066	0.6597	0.9727	0.036*	
C8	0.20156 (10)	0.62408 (10)	0.52164 (10)	0.0148 (2)	
C9	0.13809 (10)	0.58960 (10)	0.39371 (10)	0.0165 (2)	
H9	0.1258	0.6490	0.3301	0.020*	
C10	0.11971 (10)	0.39634 (10)	0.45706 (10)	0.0158 (2)	
C11	0.07801 (10)	0.27676 (10)	0.42863 (11)	0.0197 (2)	
H11A	0.0344	0.2549	0.3449	0.024*	
C12	0.10028 (11)	0.19203 (10)	0.52154 (12)	0.0221 (3)	
H12A	0.0720	0.1115	0.5021	0.027*	
C13	0.16493 (11)	0.22376 (10)	0.64566 (11)	0.0216 (2)	
H13	0.1804	0.1643	0.7095	0.026*	
C14	0.20597 (11)	0.34018 (10)	0.67553 (11)	0.0194 (2)	
H14	0.2495	0.3606	0.7597	0.023*	
C15	0.18364 (10)	0.42907 (10)	0.58167 (10)	0.0155 (2)	
H1n	0.2046 (14)	0.7886 (12)	0.4763 (11)	0.028 (4)*	
H1w	0.1780 (15)	0.9131 (16)	0.2821 (14)	0.042 (5)*	
H2w	0.0595 (11)	0.9256 (17)	0.2944 (16)	0.052 (5)*	

### Atomic displacement parameters (Å^2^)

**Table:**

	*U*^11^	*U*^22^	*U*^33^	*U*^12^	*U*^13^	*U*^23^
O1W	0.0230 (4)	0.0262 (5)	0.0170 (4)	0.0023 (3)	0.0046 (3)	0.0060 (3)
N1	0.0191 (4)	0.0137 (4)	0.0140 (4)	−0.0006 (3)	0.0010 (3)	0.0017 (3)
N2	0.0175 (4)	0.0142 (4)	0.0144 (4)	0.0003 (3)	0.0040 (3)	−0.0004 (3)
N3	0.0180 (4)	0.0180 (5)	0.0153 (5)	−0.0011 (3)	0.0042 (3)	−0.0015 (4)
C1	0.0149 (5)	0.0165 (5)	0.0170 (5)	−0.0009 (4)	0.0053 (4)	−0.0021 (4)
C2	0.0209 (5)	0.0169 (5)	0.0212 (6)	−0.0005 (4)	0.0059 (4)	−0.0002 (4)
C3	0.0238 (6)	0.0177 (5)	0.0275 (6)	−0.0040 (4)	0.0080 (5)	−0.0048 (5)
C4	0.0191 (5)	0.0254 (6)	0.0218 (6)	−0.0052 (4)	0.0058 (4)	−0.0082 (5)
C5	0.0159 (5)	0.0253 (6)	0.0162 (5)	−0.0014 (4)	0.0056 (4)	−0.0022 (4)
C6	0.0167 (5)	0.0178 (5)	0.0171 (5)	−0.0012 (4)	0.0052 (4)	−0.0004 (4)
C7	0.0211 (6)	0.0328 (7)	0.0165 (6)	−0.0019 (5)	0.0022 (4)	−0.0013 (5)
C8	0.0135 (5)	0.0152 (5)	0.0156 (5)	0.0008 (4)	0.0039 (4)	−0.0011 (4)
C9	0.0175 (5)	0.0169 (5)	0.0145 (5)	0.0000 (4)	0.0034 (4)	0.0016 (4)
C10	0.0149 (5)	0.0158 (5)	0.0175 (5)	0.0004 (4)	0.0061 (4)	−0.0007 (4)
C11	0.0192 (5)	0.0181 (5)	0.0223 (6)	−0.0024 (4)	0.0068 (4)	−0.0044 (4)
C12	0.0240 (6)	0.0146 (5)	0.0305 (6)	−0.0022 (4)	0.0120 (5)	−0.0021 (5)
C13	0.0265 (6)	0.0166 (5)	0.0248 (6)	0.0028 (4)	0.0125 (5)	0.0049 (4)
C14	0.0234 (5)	0.0178 (5)	0.0182 (5)	0.0025 (4)	0.0080 (4)	0.0007 (4)
C15	0.0161 (5)	0.0150 (5)	0.0167 (5)	0.0009 (4)	0.0065 (4)	−0.0006 (4)

### Geometric parameters (Å, °)

**Table:**

O1W—H1w	0.849 (9)	C5—C7	1.5110 (16)
O1W—H2w	0.842 (9)	C6—H6	0.9500
N1—C8	1.3625 (14)	C7—H7A	0.9800
N1—C1	1.4080 (13)	C7—H7B	0.9800
N1—H1n	0.869 (9)	C7—H7C	0.9800
N2—C8	1.3164 (14)	C8—C9	1.4480 (15)
N2—C15	1.3781 (14)	C9—H9	0.9500
N3—C9	1.2977 (14)	C10—C11	1.4098 (15)
N3—C10	1.3855 (14)	C10—C15	1.4126 (15)
C1—C6	1.3998 (15)	C11—C12	1.3697 (17)
C1—C2	1.4015 (15)	C11—H11A	0.9500
C2—C3	1.3840 (16)	C12—C13	1.4069 (17)
C2—H2	0.9500	C12—H12A	0.9500
C3—C4	1.3885 (17)	C13—C14	1.3772 (16)
C3—H3	0.9500	C13—H13	0.9500
C4—C5	1.3918 (17)	C14—C15	1.4092 (15)
C4—H4	0.9500	C14—H14	0.9500
C5—C6	1.3978 (15)		
			
H1w—O1W—H2w	105.3 (18)	C5—C7—H7C	109.5
C8—N1—C1	130.07 (9)	H7A—C7—H7C	109.5
C8—N1—H1n	114.8 (10)	H7B—C7—H7C	109.5
C1—N1—H1n	115.1 (10)	N2—C8—N1	122.51 (10)
C8—N2—C15	116.54 (9)	N2—C8—C9	121.52 (10)
C9—N3—C10	116.87 (9)	N1—C8—C9	115.97 (9)
C6—C1—C2	119.61 (10)	N3—C9—C8	122.84 (10)
C6—C1—N1	124.39 (10)	N3—C9—H9	118.6
C2—C1—N1	115.90 (10)	C8—C9—H9	118.6
C3—C2—C1	119.67 (11)	N3—C10—C11	119.55 (10)
C3—C2—H2	120.2	N3—C10—C15	120.48 (10)
C1—C2—H2	120.2	C11—C10—C15	119.98 (10)
C2—C3—C4	120.66 (11)	C12—C11—C10	120.08 (11)
C2—C3—H3	119.7	C12—C11—H11A	120.0
C4—C3—H3	119.7	C10—C11—H11A	120.0
C3—C4—C5	120.39 (10)	C11—C12—C13	120.24 (10)
C3—C4—H4	119.8	C11—C12—H12A	119.9
C5—C4—H4	119.8	C13—C12—H12A	119.9
C4—C5—C6	119.30 (10)	C14—C13—C12	120.61 (11)
C4—C5—C7	120.55 (10)	C14—C13—H13	119.7
C6—C5—C7	120.14 (11)	C12—C13—H13	119.7
C5—C6—C1	120.34 (10)	C13—C14—C15	120.20 (11)
C5—C6—H6	119.8	C13—C14—H14	119.9
C1—C6—H6	119.8	C15—C14—H14	119.9
C5—C7—H7A	109.5	N2—C15—C14	119.39 (10)
C5—C7—H7B	109.5	N2—C15—C10	121.72 (10)
H7A—C7—H7B	109.5	C14—C15—C10	118.89 (10)
			
C8—N1—C1—C6	8.48 (18)	N2—C8—C9—N3	0.87 (17)
C8—N1—C1—C2	−175.06 (11)	N1—C8—C9—N3	−178.34 (10)
C6—C1—C2—C3	1.34 (16)	C9—N3—C10—C11	−179.84 (10)
N1—C1—C2—C3	−175.31 (10)	C9—N3—C10—C15	0.27 (15)
C1—C2—C3—C4	−0.14 (17)	N3—C10—C11—C12	179.60 (10)
C2—C3—C4—C5	−1.21 (18)	C15—C10—C11—C12	−0.51 (16)
C3—C4—C5—C6	1.34 (17)	C10—C11—C12—C13	−0.07 (17)
C3—C4—C5—C7	−179.84 (10)	C11—C12—C13—C14	0.36 (18)
C4—C5—C6—C1	−0.13 (16)	C12—C13—C14—C15	−0.06 (17)
C7—C5—C6—C1	−178.95 (10)	C8—N2—C15—C14	178.78 (10)
C2—C1—C6—C5	−1.20 (16)	C8—N2—C15—C10	−2.12 (15)
N1—C1—C6—C5	175.14 (10)	C13—C14—C15—N2	178.62 (10)
C15—N2—C8—N1	−179.88 (9)	C13—C14—C15—C10	−0.51 (16)
C15—N2—C8—C9	0.96 (15)	N3—C10—C15—N2	1.58 (16)
C1—N1—C8—N2	9.51 (18)	C11—C10—C15—N2	−178.32 (10)
C1—N1—C8—C9	−171.29 (10)	N3—C10—C15—C14	−179.31 (10)
C10—N3—C9—C8	−1.45 (16)	C11—C10—C15—C14	0.79 (16)

### Hydrogen-bond geometry (Å, °)

**Table:**

Cg1 is the centroid of the C10–C15 ring.

**Table:**

*D*—H···*A*	*D*—H	H···*A*	*D*···*A*	*D*—H···*A*
C6—H6···N2	0.95	2.34	2.9482 (14)	122
N1—H1n···O1w	0.87 (1)	2.03 (1)	2.8951 (12)	176.(2)
O1w—H1w···N2^i^	0.85 (1)	2.13 (1)	2.9382 (13)	160.(2)
O1w—H2w···N3^ii^	0.84 (1)	2.15 (1)	2.9504 (12)	158.(2)
C7—H7b···Cg1^iii^	0.98	2.71	3.6532 (14)	161

Symmetry codes:  (i) *x*, −*y*+3/2, *z*−1/2; (ii) −*x*, *y*+1/2, −*z*+1/2; (iii) −*x*+1, *y*+1/2, −*z*+3/2.
